# Isolated open tibial shaft fracture: a seven-hospital, prospective observational study in two Latin America countries

**DOI:** 10.1590/0100-6991e-20223301-en

**Published:** 2022-11-10

**Authors:** WILLIAM DIAS BELANGERO, FABRICIO FOGAGNOLO, KODI EDSON KOJIMA, GUILHERME CHOHFI DE MIGUEL, FERNANDO BIDOLEGUI, ALEJANDRO DANIEL BERTUNE, ERNESTO LOMBARDO, ADÉLIO DE LIMA DIAS, JOÃO BATISTA MANZOLI TORRES, BRUNO PARILHA COUTINHO, JORGE DOS SANTOS SILVA, MARCOS DE CAMARGO LEONHARDT, PABLO SEBASTIÁN PEREIRA, JOSÉ RICARDO LENZI MARIOLANI, VINCENZO GIORDANO

**Affiliations:** 1 - Universidade Estadual de Campinas, Faculdade de Ciências Médicas - Campinas - SP - Brasil; 2 - Universidade de São Paulo, Faculdade de Medicina - Ribeirão Preto - SP - Brasil; 3 - Universidade de São Paulo, Instituto de Ortopedia e Traumatologia - São Paulo - SP - Brasil; 4 - Universidade São Francisco, Hospital Universitário São Francisco - Bragança Paulista - SP - Brasil; 5 - Hospital Sirio Libanes - Buenos Aires - Argentina; 6 - Hospital de Emergencias Clemente Alvarez - Rosario - Santa Fé - Argentina; 7 - Hospital Municipal Miguel Couto, Serviço de Ortopedia e Traumatologia Prof. Nova Monteiro - Rio de Janeiro - RJ - Brasil

**Keywords:** Fractures, Open, Tibial Fracture, Return-to-Work, Treatment Outcome, Fracture Healing, Fraturas Expostas, Fraturas da Tíbia, Desfecho do Tratamento, Retorno ao Trabalho, Consolidação da Fratura

## Abstract

**Introduction:**

open tibial fractures are challenging due to the frequent severe bone injury associated with poor soft tissue conditions. This is relevant in low- and middle-income countries, mainly related to delayed definitive fixation and lack of adequate training in soft tissue coverage procedures. Due to these factors, open tibial fracture is an important source of disability for Latin American countries. Herein we sought to provide an epidemiological overview of isolated open tibial shaft fracture across seven hospitals in southern cone of Latin America. The secondary goal was to assess the impact on quality of life based on return-to-work rate (RWR).

**Methods:**

patients with an isolated open tibial shaft fracture treated in seven different hospitals from Brazil and Argentina from November 2017 to March 2020 were included in the study. Clinical and radiographic results were evaluated throughout the 120-day follow-up period. Final evaluation compared RWR with the SF-12 questionnaire, bone healing, and gait status.

**Results:**

Seventy-two patients were treated, 57 followed for 120 days and 48 completed the SF-12 questionnaire. After 120 days, 70.6% had returned to work, 61.4% had experienced bone healing. Age, antibiotic therapy, type of definitive treatment, and infection significantly influenced the RWR. Gait status exhibited strong correlations with RWR and SF-12 physical component score.

**Conclusions:**

Isolated open tibial shaft fractures are potentially harmful to the patient’s quality of life after 120 days of the initial management. RWR is significantly higher for younger patients, no history of infection, and those who could run in the gait status assessment.

## RESUMO

**Introdução:**

o tratamento de fraturas expostas isoladas da diáfise da tíbia (FEIDT) apresenta desafios por frequentemente associar severa lesão óssea com condições ruins de tecido mole, fatores relevantes em países de média e baixa renda, especialmente devido a atrasos na implementação da fixação definitiva e falta de treinamento adequado no manejo de tecidos moles. Consequentemente, FEIDTs representam importante fonte de incapacitação na América Latina. Este estudo objetivou apresentar uma visão geral das FEIDTs em sete hospitais do cone sul da América Latina. O objetivo secundário foi avaliar o seu impacto na qualidade de vida baseado na taxa de retorno ao trabalho (TRT).

**Métodos:**

foram incluídos no estudo pacientes com FEIDT tratados em sete hospitais de Brasil e Argentina entre novembro de 2017 e março de 2020. Resultados clínicos e radiográficos foram analisados num período de 120 dias. Avaliação final comparou TRT com o questionário SF-12, consolidação óssea e condições de marcha.

**Resultados:**

setenta e dois pacientes foram tratados, 57 seguidos por 120 dias e 48 completaram o questionário SF-12. Após 120 dias, 70,6% havia retornado ao trabalho, 61,4% tinha fratura consolidada. Idade, antibioticoterapia, tipo de tratamento definitivo e infecção influenciaram significativamente na TRT. A condição de marcha apresentou forte correlação com TRT e o componente físico do SF-12.

**Conclusão:**

FEIDTs são potencialmente deletérias à qualidade de vida dos pacientes 120 dias após o tratamento inicial. TRT é significativamente maior para pacientes mais jovens, sem história de infecção e que conseguem correr na avaliação da condição de marcha..

## INTRODUCTION

Tibia fracture is the most common long bone fracture, with severe soft tissue injury and open fractures occurring in up to 24% of cases^1^ ^-^ ^3^. Despite advances in medical care and treatment, the incidence of complications remains worryingly high, with reported reoperation rates ranging from 4% to 48%^4^.

This is particularly critical in low- and middle-income countries (LMICs) in Latin America, especially due to the delayed definitive fixation and the lack of adequate training in soft tissue coverage procedures, which potentially increase the risk of fracture-related infection. Severe local infection is directly related to the presence of major soft tissue trauma. A recent study from the Asociación de Cirujanos Traumatólogos en las Americas (ACTUAR) Open Tibia Study Group showed that few Latin American countries had available guidelines for the treatment of open tibial shaft fractures^5^. The lack of formal guidelines combined to the increased number and severity of road traffic accidents have burdened the health systems and increased costs related to the treatment of these patients in Latin America.

Considering to better understanding the treatment of isolated tibial fractures, AO Trauma Latin America (AOTLA) sponsored a prospective observational study in these two countries. Therefore, in the herein study, we sought to provide an epidemiological overview of isolated open tibial shaft fracture across seven hospitals in southern cone of Latin America. The secondary goal was to assess the impact on quality of life based on return-to-work rate (RWR), SF-12 and gait status.

## METHODS

Elegible patients with an isolated open tibial shaft fracture treated in seven different hospitals from Brazil and Argentina from November 2017 to March 2020 were included in the study. In Brazil, there were four public hospitals (three of them university hospitals) and one private hospital (a university hospital), and in Argentina there were one public hospital and one private hospital. Ethics approval was obtained from all local authorities and the Institutional Review Board was located at the School of Medical Sciences, University of Campinas, Brazil. Approval of the project is identified as CAAE Approval 1.685.971, August 19, 2016. The study was conducted in accordance with the ethics principles outlined in the 2016 Declaration of Helsinki and followed the local laws and regulations of Argentina and Brazil. All patients enrolled in the study or the patient responsible signed the Informed Consent Form (ICF).

Inclusion criteria were age ≥18 years; presence of an isolated open tibia shaft fracture; no previous treatment of this injury; patient’s ability to understand and sign the ICF (or be represented by a legally authorized, responsible person); and patient’s agreement to participate in the study in accordance with the approved protocol. All subjects were recruited from emergency departments by the participating centers. Exclusion criteria were pathological fractures; polytrauma; previous fractures in the same bone with sequela that might affect treatment of the new fracture; metabolic bone disease; incomplete clinical history; any treatment performed at some other hospital not involved in the study; neurological and/or psychiatric disorders that prevented a reliable evaluation (e.g., Parkinson’s disease, multiple sclerosis, severe depression); and limited freedom (e.g., arrested patients).

Treatment protocol and postoperative medical care were chosen according to the surgeon’s preference and the guidelines of each participating hospital. At hospital admission, demographic data (age, gender, occupational status, presence of chronic diseases, cigarette smoking, and alcohol use) and fracture-generating event data (date, time, mechanism of injury, and alcohol and/or illicit drug use) were collected. Data collected at the time of initial treatment included antibiotic therapy, initial treatment and definitive closure of the soft tissue injury, initial bone injury treatment, presence/absence of compartment syndrome, AO/OTA^6^ and Gustilo et al.^7^ classifications, and American Society of Anaesthesiologists (ASA)^8^ classification.

During follow-up, data were collected at regular time intervals between hospital admission and the final evaluation following a predefined protocol.

After hospital discharge, patients were strictly followed on the outpatient clinic of the hospital enrolled in the study. All patients were evaluated at 30, 60, 90, and 120 days after definitive treatment. A web-based electronic data capture system sponsored by AOTLA was created for the inclusion of the data and participating surgeons entered all data into the system at these time intervals. Surgeons were previously registered with assigned login and password credentials to be able to add data of their patients. A number to ensure their privacy and anonymity of their data identified each patient.

After 120 days, patients were comprehensively evaluated and asked to complete the 12-Item Short Form Health Survey (SF-12) questionnaire^9^ ^,^ ^10^. During radiographic evaluation, a fracture was considered healed if there was callus formation at no fewer than three cortices on orthogonal views^11^ ^-^ ^12^. The same surgeon who conducted the surgical procedure performed this evaluation.

The main outcome was return-to-work rate (RWR). Additionally, the SF-12 physical and mental components, bone healing, and gait status were assessed. The Portuguese version of the SF-12 used in the study had been validated in Brazil in a population with chronic obstructive pulmonary disease^13^. The Spanish version of the SF-12 was adapted and validated in Spain^14^. The criteria used to gait status evaluation was proposed by Macri et al.^15^.

Categorical data were analyzed using Pearson’s χ2 analysis. The student’s t-test was employed to compare normally distributed continuous variables when there were only two groups, while analysis of variance (ANOVA) was used for more than two groups. For non-normally distributed continuous variables, the Mann-Whitney U-test was used for two groups and the Kruskal-Wallis test, with the Bonferroni-Sidak test employed post hoc for three or more groups. The degree and statistical significance of any correlation between two continuous variables were determined by generating a Spearman’s rank correlation coefficient (ρ). All results were considered significant at p<0.05. The statistical software used was SPSS 15.0 for Windows (SPPS Inc., Chicago, IL, USA).

## RESULTS

**Figure 1:**
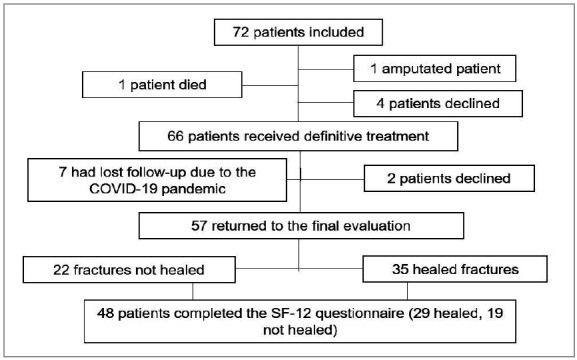
Flowchart of the study..

Seventy-two patients were enrolled between November 2017 and March 2020, 62 from Brazil and 10 from Argentina. Of these, 57 patients were followed for 120 days and 48 completed the SF-12 questionnaire. Figure 1 is the study flowchart.

Table 1 shows the demographic and fracture characteristics of the 72 patients included in the study, and of the 57 patients who reached the 120-day evaluation. The characteristics of the two groups remained quite similar, except that all the patients lost to follow-up were men.

**Table 1:** Comparing demographic, fracture-related, and initial treatment-related characteristics in the 72 patients initially enrolled and 57 patients who completed 120 days of follow-up..

Characteristic	Number of patients (%)	
	Enrolled (n=72)	Completed follow-up (n=57)
Demographic characteristics		
Sex		
Male	61 (84.7%)	46 (80.7%)
Female	11 (15.3%)	11 (19.3%)
Age group (years)		
18-40	50 (69.4%)	37 (64.9%)
41-65	20 (27.8%)	19 (33.3%)
>65	2 (2.8%)	1 (1.8%)
Professional activity		
Motorcycle courier	16 (22.2%)	12 (21.1%)
Workman	20 (27.8%)	15 (26.3%)
Unemployed, student, retired	11 (15.3%)	10 (17.5%)
Other	25 (34.7%)	20 (35.1%)
Smoker		
No	62 (86.1%)	48 (84.2%)
Yes	10 (13.9%)	9 (15.8%)
Alcohol drinker		
Characteristic	Number of patients (%)	
	Enrolled (n=72)	Completed follow-up (n=57)
No	23 (32.0%)	21 (36.9%)
Occasionally	43 (59.7%)	30 (52.6%)
Daily	6 (8.3%)	6 (10.5%)
Chronic disease		
No	58 (80.6%)	44 (77.2%)
Yes	14 (19.4%)	13 (22.8%)
Characteristics related to the fracture		
Mechanism of injury		
Motorcycle accident	43 (59.7%)	33 (57.9%)
Non-motorcycle traffic accident	13 (18.1%)	10 (17.5%)
Other	16 (22.2%)	14 (24.6%)
Under the influence of alcohol/drugs when the fracture occurred		
No	62 (86.1%)	50 (87.7%)
Yes	10 (13.9%)	7 (12.3%)
AO classification		
42A	37 (51.4%)	31 (54.4%)
42B	24 (33.3%)	16 (28.1%)
42C	11 (15.3%)	10 (17.5%)
Gustilo et al. classification		
I	9 (12.5%)	5 (8.8%)
II	23 (32.0%)	22 (38.6%)
III A	33 (45.8%)	26 (45.6%)
III B and C	7 (9.7%)	4 (7.0%)
Patient ASA classification		
I	52 (72.2%)	39 (68.4%)
II	17 (23.6%)	16 (28.0%)
III	0 (0.0%)	0 (0.0%)
IV	2 (2.8%)	1 (1.8%)
No response	1 (1.4%)	1 (1.8%)
Characteristics of the initial treatment		
Antibiotic therapy		
Cephalosporin only	17 (23.6%)	13 (22.8%)
Cephalosporin + Aminoglycoside	14 (19.4%)	11 (19.3%)
Cephalosporin + Aminoglycoside + Metronidazole	23 (32.0%)	17 (29.8%)
Gentamicin + Clindamycin	10 (13.9%)	10 (17.5%)
Other combinations	8 (11.1%)	6 (10.5%)
Characteristic	Number of patients (%)	
	Enrolled (n=72)	Completed follow-up (n=57)
Closure of soft tissue injury		
Primary closure	53 (73.6%)	42 (73.7%)
Partial closure or wound left open	19 (26.4%)	15 (26.3%)
Soft tissue injury treatment		
Debridement	61 (84.7%)	51 (89.5%)
Other	11 (15.3%)	6 (10.5%)
Initial bone injury treatment		
External fixation	42 (58.3%)	35 (61.4%)
Intramedullary nail	19 (26.4%)	13 (22.8%)
Bridge plate	4 (5.6%)	4 (7.0%)
Other	7 (9.7%)	5 (8.8%)
Patient had compartment syndrome		
No	71 (98.6%)	56 (98.2%)
Yes	1 (1.4%)	1 (1.8%)

Note that the 57 patients who completed four months of follow-up are included among the 72 initially enrolled.

Of the 57 patients who reached the final evaluation, the mean age was 37.3 ± 10.9 years [mean ± standard deviation (SD)] among men, 39.5 ± 21.1 years among women, and 37.8 ± 13.4 years overall. No significant influence of gender, professional activity, smoking habit, alcohol use, or presence of chronic disease was found.

The average time from hospital admission to the initial surgery was 6.8 hours (range = 0 to 46 hours) and from hospital admission to the initiation of antibiotic therapy was 2.1 hours (0 to 12.5h). At the operating room (OR), the mean duration of wound irrigation and debridement was 1.4 hours (0.17 to 3.0h).

Table 2 brings the comparison between patients’ RWR, fracture status on radiographic imaging (healed vs. not healed), and SF-12 questionnaire physical and mental component scores for different study variables.

**Table 2:** SF-12 scores (PCS and MCS), fracture status and return-to-work rate (RWR) after 120 days for different study variables.

Characteristic	SF-12 scores (n=48)				Fracture status (n=57)					RWR (n=51)
	PCS		MCS							
	Median (IQR)	p	Median (IQR)	p	Healed	Not healed	p	Yes	No	p
Age of the patients in years										
[mean (standard deviation)]	-	-	-	-	35.1 (13.3)	40.3 (13.0)	0.150^e^	35.2 (12.9)	43.7 (14.4)	0.044^e^^*^
Characteristic	SF-12 scores (n=48)				Fracture status (n=57)					RWR (n=51)
	PCS		MCS							
	Median (IQR)	p	Median (IQR)	p	Healed	Not healed	p	Yes	No	p
Mechanism of injury										
Motorcycle accident	48.4 (17.9)	0.240^b^	56.9 (16.3)	0.941^b^	20	13	0.159^d^	23	7	
Non-motorcycle traffic accident	37.5 (25.0)		55.1 (4.7)		4	6		4	4	0.336^d^
Other	48.1 (10.7)		56.8 (8.3)		11	3		9	4	
AO classification										
42A	45.9 (15.3)	0.944^b^	57.4 (7.8)	0.273^b^	18	13	0.379^d^	20	8	
42B	45.9 (20.5)		55.1 (7.0)		12	4		10	4	0.960^d^
42C	48.4 (20.6)		53.1 (15.9)		5	5		6	3	
Gustilo et al. classification										
I	47.9 (20.7)	0.580^b^	48.9 (13.8)	0.622^b^	4	1	0.669^d^	3	1	0.290^d^
II	40.7 (15.6)		57.8 (18.1)		12	10		10	8	
IIIA	49.0 (11.9)		55.7 (6.7)		17	9		20	6	
IIIB and C	37.0 (11.4)		59.0 (8.0)		2	2		3	0	
Patient ASA classification (n=56)										
I	48.4 (18.4)	0.906^b^	55.7 (14.9)	0.866^b^	22	17	0.501^d^	21	13	
II	45.9 (17.3)		56.2 (8.2)		11	5		13	2	0.173^d^
IV	51.7 (0.0)		58.7 (0.0)		1	0		1	0	
Time (hours) from hospital admission to surgery initiation [median (interquartile range)]				3.0 (2.0)		6.5 (14.0)	0.060^a^	3.0 (3.3)	4.0 (10.0)	0.199^a^
Time (min) from hospital admission to antibiotic therapy initiation [median (interquartile range)]				87.9 (60.0)		186.5 (179.3)	0.339^a^	52.5 (67.8)	60.0 (70.0)	0.403^a^
Length (min) of the procedure [mean (standard deviation)]				81.4 (38.5)		94.3 (35.7)	0.209^e^	86.1 (39.1)	95.7 (34.7)	0.454^e^
Antibiotic therapy										
Cephalosporin only	39.7 (14.1)	0.024^b^	53.6 (18.2)	0.064^b^	8	5	0.195^d^	4	5	0.021^d^
Cephalosporin/Aminoglycoside	51.7 (16.6)	*	56.9 (3.9)		8	3		9	1	*
Cephalosporin/Aminoglycoside/Metronidazole^1^	40.2 (12.3)	Post hoc	59.2 (7.0)		11	6		9	8	
Characteristic	SF-12 scores (n=48)				Fracture status (n=57)					RWR (n=51)
	PCS		MCS							
	Median (IQR)	p	Median (IQR)	p	Healed	Not healed	p	Yes	No	p
Gentamicin/ Clindamycin^2^	55.1 (3.0)	1x2:	59.7 (6.4)		7	3		9	0	
Other combinations	45.2 (14.0)	0.037^c^	52.2 (12.3)		1	5		5	1	
Closure of soft tissue injury										
Primary closure	48.7 (15.7)	0.098^a^	55.1 (16.4)	0.248^a^	26	16	0.987^d^	26	10	0.692^d^
Partial closure or wound left open	37.9 (16.7)		57.4 (6.9)		9	6		10	5	
Soft tissue injury treatment										
Debridement	45.9 (17.5)	0.761^a^	55.1 (11.2)	0.661^a^	32	19	0.544^d^	30	15	0.092^d^
Other	48.4 (15.8)		56.9 (4.0)		3	3		6	0	
Initial bone injury treatment										
External fixation	42.0 (15.9)	0.085^b^	57.8 (13.4)	0.671^b^	23	12	0.687^d^	22	9	0.060^d^
Intramedullary nail	55.2 (7.6)		55.1 (6.1)		7	6		11	1	
Bridge plate	42.4 (6.9)		46.1 (12.1)		2	2		1	3	
Other	42.2 (8.7)		57.7 (6.7)		3	2		2	2	
Wound condition at definitive treatment										
Without infection	44.8 (17.6)	0.306^a^	55.7 (10.3)	1.000^a^	30	17	0.415^d^	35	10	0.002^d^
At the risk of being infected	52.1 (10.3)		56.9 (6.6)		5	5		1	5	*
Soft tissue injury at definitive treatment										
No need of additional care	45.9 (15.7)	0.833^a^	55.1 (11.3)	0.853^a^	31	17	0.255^d^	29	14	0.253^d^
Local flap/other	48.4 (22.8)		56.9 (4.3)		4	5		7	1	
Days between initial and definitive treatment [median (interquartile range)]					5.0 (10.0)	5.5 (10.0)	0.725^a^	5.5 (10.3)	4.0 (8.5)	0.462^a^
Treatment of bone injury										
Intramedullary nailing	47.2 (17.5)		55.7 (8.3)	0.630^b^	29	17		34	6	<0.001^d^
Plate	44.4 (12.4)	0.574^b^	58.0 (8.1)		4	4	0.769^d^	2	6	*
Characteristic	SF-12 scores (n=48)				Fracture status (n=57)					RWR (n=51)
	PCS		MCS							
	Median (IQR)	p	Median (IQR)	p	Healed	Not healed	p	Yes	No	p
External fixator	34.9 (0.0)		46.7 (0.0)		2	1		0	3	
Cortical contact at the time of definitive treatment				0.005^b^ *						
>95%^1^	49.0 (15.9)	0.359^b^	57.9 (6.7)	Post hoc	20	9	0.012^d^ * _	19	7	
65-95%	44.8 (13.3)		55.1 (5.6)	1x2:	15	8		15	6	0.637^d^
<65%^2^	48.4 (32.9)		39.3 (2.7)	0.017c	0	5		2	2	
Diastasis at fracture site										
No	47.8 (16.8)	0.436^a^	56.6 (6.9)	0.383^a^	32	18	0.282^d^	32	13	0.637^d^
Yes	39.2 (25.5)		43.6 (17.2)		3	4		4	2	

IQR: inter-quartile range; ^a^Mann-Whitney U test; ^b^Kruskal-Wallis test; ^c^Bonferroni-Sidak post hoc test; ^d^Pearson’s chi-square analysis; ^e^Student’s t test; *Statistically-significant difference; “Post hoc i x j:0.0xx” = post hoc comparison of group i versus group j; p=0.0xx.

After 120 days, 35 (61.4%) fractures had healed. 51 patients reported to be working before the fracture, with 36 (70.6%) reported having returned to work by day 120 of follow-up. Those who returned to work were significantly younger (a mean 8.5 years) compared to those who had not. There was a strong correlation between RWR and gait status (p<0.001). Of the 16 patients who walked with a limp, 14 did not return to work, while of the nine who walked without a limp, only one did not return to work. The percentage of cortical contact at the time of definitive surgery was found to be related to bone healing status. The physical component score (PCS) of the 48 patients who completed the SF-12 questionnaire ranged from 20.8 to 60.5, with mean of 44.5 and SD of 11.2, while the mental component score (MCS) ranged from 31.2 to 66.4 (53.5±8.9).

Correlations were sought between patients’ SF-12 scores and patient age, time from hospital admission to the initiation of antibiotic therapy to surgery, length of the surgical procedure, time with external fixation, and time between the initial and the definitive treatment. Very week correlations were detected between the PCS and the time from hospital admission to surgery (Spearman’s ρ=-0.386, p=0.007), time between initial and definitive treatment (ρ=-0.357, p=0.013), and the surgeon’s bone injury treatment evaluation (ρ=0.316, p=0.029). In addition, very week correlation was detected between the MCS and patient’s age (ρ=-0.291, p=0.045).

Table 3 compares the different outcomes assessed in this study. Both the physical and mental component scores were significantly higher among patients with fracture healing versus those without, those who had returned to work versus not, and those with no limp. The mean ± SD of the PCS among patients with healed fractures was 48.29±8.14, versus 38.73±12.90 in patients whose fracture did not heal. Comparable MCS were 56.30±6.55 and 49.28±10.41, respectively. The PCS also was higher among patients who rated themselves better upon self-evaluation. Wound status (infected vs. not) had no influence on either score.

**Table 3:** Comparing the different outcomes assessed in this study.

Characteristic	SF-12 scores (n=48)				Fracture status (n=57)				RWR (n=51)	
	PCS		MCS							
	Median (IQR)	p	Median (IQR)	p	Healed	Not healed	p	Yes	No	p
SF-12 Physical component score (PCS)	-	-	-	-	49,4 (13,6)	39,7 (21,4)	0,006^a^ *	52,3 (11,0)	29,2 (15,9)	<0,001^a^ *
SF-12 Mental component score (MCS)	-	-	-	-	57,9 (5,5)	46,7 (15,0)	0,015^a^ *	57,9 (5,7)	49,8 (11,0)	0,010^a^ *
Characteristic	SF-12 scores (n=48)				Fracture status (n=57)				RWR (n=51)	
	PCS		MCS							
	Median (IQR)	p	Median (IQR)	p	Healed	Not healed	p	Yes	No	p
Fracture Status										
Healed	49,4 (13,6)	0,006^a^	57,9 (5,5)	0,015^a^	35	-	-	27	5	0,005^d^
Not healed	39,7 (21,4)	*	46,7 (15,0)	*	-	22		9	10	
Return-to-work										
Yes	52,3 (11,0)	<0,001^a^	57,9 (5,7)	0,010^a^	27	9	0,005^d^	36	-	-
No	29,2 (15,9)	*	49,8 (11,0)	*	5	10		-	15	
Patient’s gait status		0,001^b*^								
Patient walks with a limp^1^	33,2 (14,8)	Post hoc	46,7 (14,6)	0,049^b^	5	11		2	14	
Patient walks without limping^2^	47,8 (10,0)	1x4: 0,045^c^	53,8 (14,6)	*	3	8		8	1	
Patient walks fast without limping^3^	41,9 (7,2)	1x5: 0,031^c^	60,2 (6,5)	Post hoc	6	2	< 0,001^d^ *	6	0	<0,001^d^ *
Patient runs with a limp^4^	55,2 (10,6)	2x5: 0,047^c^	58,1 (5,9)	1x5:	7	1		8	0	
Patient runs without limping5	55,5 (3,1)	3x5: 0,011^c^	57,9 (1,9)	0,049^c^	11	0		12	0	
Wound healing										
Without infection	48,4 (17,4)	0,175^a^	56,2 (9,2)	0,842^a^	29	19	0,624^d^	35	10	0,002^d^
With signs of infection	36,2 (26,2)		55,9 (7,6)		3	3		1	5	*

IQR: intervalo interquartil; ^a^teste U de Mann-Whitney; ^b^Teste de Kruskal-Wallis; ^c^teste post hoc de Bonferroni-Sidak; ^d^Teste Qui-quadrado de Pearson; *Diferença estatisticamente significativa; “Post hoc i x j:0,0xx” = comparação post hoc do grupo i versus grupo j; p=0,0xx.

The outcomes were compared between patients who underwent definitive treatment with intramedullary (IM) nailing, plating, or external fixation, with the only difference being the gait status. 16% of patients undergoing IM nailing had a limp after 120 days compared to 75% of patients undergoing plating and 100% of patients undergoing external fixation (p=0.008).

Six patients had fracture-related infection, 4 in patients originally presenting a Gustilo et al. IIIA-grade fracture. These patients were initially treated with intravenous cephalosporin and aminoglycoside. No significant difference was detected in the time of initiation of the antibiotic drug between patients with (median = 60.0 min, interquartile range = 38.5min) and without (60.0min, IQ=92.5min) a postoperative fracture-related infection (p=0.93, Mann-Whitney U test).

## DISCUSSION

The RWR was the most important outcome, and it was significantly influenced by the patient’s age, the presence of a wound infection, and the type of definitive treatment. A younger age favored the return to work, but not fracture healing, contrary to the results reported by Yusof et al.^16^, who found a significant difference in the mean age of Malay patients who had fracture healing (27.5 years) compared to those who did not (40.3 years). Moreover, Leliveld et al.^17^ identified correlations between older age and longer hospital lengths of stay and years living with disability in Dutch patients with isolated tibial shaft fractures. In our study, 38.6% of the fractures were not healed at the time of final evaluation, which can be attributed to the short time of follow-up (120 days). In a systematic review involving 111 studies and 41,429 patients with all kinds of tibial fracture, Tian et al. observed non-union rates ranging from 0% to 42.7%^18^.

Definitive fixation with an IM nail proved to be significantly superior to a plate or external fixator. However, this result should be taken with caution, given the small number of patients treated with external fixator (n=3) or plate (n=8). Although several authors demonstrated better results using IM nails compared to external fixators, similar clinical results have been shown between IM nailing and minimally invasive plating osteosynthesis for the treatment of open fractures of the tibial shaft^19^ ^-^ ^22^. DiSilvio et al. found that the presence of any bridging callus after four months of surgery predicts accurately that tibia fractures treated with IM nail will finally heal^23^. In our study, of the 22 patients who did not present consolidation at 120 days, 21 (95.4%) had at least one bridging callus.

After 120 days, 29.4% of our patients who were working prior to the fracture had not returned to work. In a systematic review including 14 studies (n=795), about 60% of patients fully returned to work, 17% returned to part-time work or had to change occupation and 22% did not return to work after one year of follow-up^24^. Some of these studies reported higher RWR due to country-specific rehabilitation programs and some reported lower RWR due to focus on physical demanding professions. In our study, the professional activity did not significantly influence the RWR. It is interesting to note that the presence of a wound infection significantly influenced the RWR, but not the rate of bone healing. There were patients who returned to work without fracture healing, and patients who did not return even with their fracture healed and no signs of infection. This highlights the need for cautiously interpret the RWR as the sole predictor of outcome. Conversely, gait assessment proved to be an excellent parameter to assess patient’s recovery. We found that all patients who could walk fast or run after 120 days had already returned to work, whereas 14 (87.5%) of 16 patients who walked with a limp did not return to work. Gait assessment eliminates much of the subjectivity present in both the SF-12 questionnaire or the radiographic assessment of bone healing.

The SF-12 was chosen because this assessment questionnaire proved to be easy and fast, being completed in less than 2 minutes by about 80% of adults^25^. In the present study, 94% of the respondents were from Brazil, with a mean PCS value of 44.5 and a mean MCS of 53.5. In a random sample of 5,000 Brazilian citizens, Campolina et al. obtained a mean of 49.3 for the PCS and 52.7 for the MCS^26^. Our results are very similar to those from Tay et al., who failed to detect any influence of diaphyseal fractures of the femur or tibia on patients’ mental state and inferred that the preliminary effects of these injuries during the recovery phase are predominantly physical^27^. It is noteworthy that the lowest MCS values in our study were observed in patients treated with an external fixation as definitive treatment, who had less than 65% cortical contact immediately after definitive surgery, or who were unable to achieve bone healing at final follow-up after 120 days. Ultimately, the median MCS of patients with low cortical contact was significantly lower than was that of patients with more than 95% contact.

The mean age of the patients included in this study (37.5 years) was very close to the 35 years found in a systematic review covering low- and middle-income countries (LMICs) from Asia and Africa^28^, and the 38.5-year-old average observed in a Danish study^29^.

Other European studies and one conducted in Singapore detected slightly higher average ages, between 43.3 and 47.0 years old^30^ ^-^ ^33^. In our study, 84.7% of the patients were male, very close to the 87% reported by Schade et al.^28^. In other studies, the proportion of men was consistently lower, ranging from 63.8% to 72.4%^29^ ^-^ ^33^. Although the literature points towards a predominance of young men and elderly women among the victims of open tibial fractures, this distribution was not observed in our study^31^ ^,^ ^34^. The lower mean age and higher proportion of men in our study may be a consequence of the more frequent use of motorcycles among young men, mainly in Brazil, but also in Argentina^35^. 59.7% of our patients fractured their tibia in a motorcycle accident, against 22.9% in Weber et al.^30^ and 32.4% in Decruz et al.^32^ (no specific data about motorcycle accidents are reported for the other, previously cited studies). We found 55.5% Gustilo et al. type III fractures in our study, which was very similar to the numbers reported in studies conducted in Malaysia (56.9%) and Singapore (59.2%), although higher than observed in Asia and Africa (41%) and Europe (23.2% to 44.6%)^2^ ^,^ ^16^ ^,^ ^28^ ^,^ ^30^ ^,^ ^32^.

This study was primarily designed to provide an epidemiological overview of isolated open tibial fractures treated in level-1 trauma hospitals from Latin America. However, only surgeons from Brazil and Argentina accepted our invitation, with seven hospitals enrolled in the study. Although this could be seen as a limitation of the study, our findings showed a significant correlation between RWR and gait status, and a trend towards correlation between PCS and the time from hospital admission to surgery. After 120 days, patients who returned to work were on average 8.5 years younger than those who did not. Unfortunately, the study was hampered by the emergence of the COVID-19 pandemic, which forced its premature closure and resulted in the loss of patients who did not return for final evaluation.

Nevertheless, although 72 patients were enrolled in the study, 57 were completely followed for 120 days, what corresponds to approximately 20% loss to follow-up. Noteworthy, only male patients failed to complete follow-up, almost all of them from 18 to 40 years old. There is much confusion when calculating how much the accepted loss to follow-up is, and when this becomes problematic, leading to bias. It is generally considered that <5% loss leads to little bias, while >20% poses serious threats to validity in randomized controlled trials, however there is no clear recommendation in prospective, observational studies^36^. Therefore, it is critical to determine which mechanism generated the loss to follow-up in these types of study.Kristman et al. recognized three mechanisms of loss to follow-up: missing completely at random, missing at random, and missing not at random^37^. These authors found no important bias with levels of loss that varied from 5 to 60% when loss to follow-up was related to missing completely at random or missing at random mechanisms. In our study, all patients who did not complete the 120-day follow-up were lost in a non-random way, due to either treatment dropout or non-attendance on the correct date scheduled for follow-up, which motivated their exclusion for non-compliance with the protocol.

Another potential limitation of the study is the risk of type I error, inherent when analyzes involve multiple comparisons. Due to the premature termination of the study, it was not possible to perform the Bonferroni p-value adjustments, which could show the statistical power to detect differences. Howsoever, the characteristics of the group did not change significantly. Furthermore, the aim of our study was to be exploratory rather than testing any hypothesis.

On the other hand, we believe that our study has several strengths. Working with seven hospitals, in close communication with all surgeons involved in the study, performing a detailed collection of patient data, and using a comprehensive and easy-to-handle database should all be seen as positive aspects of the study. There were five hospitals from Brazil and two from Argentina, all level-1 trauma centers, and the aim was to assess the impact of isolated injury, without other influencing factors, on return to normal activities and quality of life, again with no intention of testing any formal hypothesis. The epidemiological studies of tibial fractures previously available in the literature have generally been less restrictive to patients with multiple fractures or polytrauma patients. This makes it difficult to compare the results of our study with those of other studies on tibial fractures. Moreover, to the best of our knowledge, this is the first study to offer a broad epidemiological panorama and outcomes of isolated open tibial shaft fractures from two LMICs in Latin America.

## CONCLUSION

Isolated open tibial shaft fractures are potentially harmful to the patient’s quality of life after 120 days of the initial management. RWR is significantly higher for younger patients, no history of infection, and those who could run or walk fast in the gait status assessment. Larger and inferential studies, with targeted independent and dependent variables, will allow our findings to help in the creation of strict and reproducible guidelines for the treatment of open tibial shaft fractures throughout the Latin American region.
